# Van der Woude syndrome- a syndromic form of orofacial clefting

**DOI:** 10.4317/jced.50559

**Published:** 2012-04-01

**Authors:** R. Sudhakara Reddy, T. Ramesh, N. Vijayalaxmi, R. Lavanya Reddy, L A. Swapna, T. Rajesh Singh

**Affiliations:** 1 Professor & head of the department, Dept. Oral medicine & Radiology, Vishnu dental college, Bhimavaram; 2 Associate professor, Dept. Oral medicine & Radiology. Vishnu dental college, Bhimavaram; 3 Post graduate student, Dept. Oral medicine & Radiology, Vishnu dental college, Bhimavaram; 4 Senior lecturer, Dept. Oral medicine & Radiology, Vishnu dental college, Bhimavaram

## Abstract

Van der Woude Syndrome is the most common form of syndromic orofacial clefting, accounting for 2% of all cases, and has the phenotype that most closely resembles the more common non-syndromic forms. The syndrome has an autosomal dominant hereditary pattern with variable expressivity and a high degree of penetrance with cardinal clinical features of lip pits with a cleft lip, cleft palate, or both. This case report describes van der Woude syndrome in a 19 year old male patient with a specific reference to the various aspects of this condition, as clinical appearance, etiological factors (genetic aspects), differential diagnosis, investigative procedures and management.

** Key words:**Cleft palate, cleft lip, lip pits, van der Woude syndrome, syndromic clefting.

## Introduction

Orofacial clefting (OFC) is a common developmental genetic disorder that occurs with a prevalence which has been estimated at between 1 in 2500 live births depending on geographic origin, racial and ethnic variation, and socioeconomic status(1). van der Woude Syndrome (VWS) is the most common form of syndromic Orofacial clefting accounting for 2% of all cases (2). Lower lip pit(s) and cleft lip and palate are the cardinal features of this syndrome, having a prevalence rates varying from 1: 100000 to 1: 400000 still born or live births with equal sex predilection (3).

Etiology of this syndrome is remarkably variable, it was initially mapped to human chromosome 1q 32 – q 41 and later demonstrated to result from mutation in gene encoding Interferon Regulatory Factor 6 [IRF 6] (2). Obligate carriers of this dominant mutation may have lip pits alone, cleft(s) alone, clefts and pits, or neither (3). OFC may occur as part of a syndrome, where structures other than the lip and palate are affected; over 70% of cases of cleft lip and 50% of cases of cleft palate arise in the absence of other abnormalities and are collectively classified as non-syndromic (2). Individuals who exhibit OFC may experience problems with eating, speaking, hearing and facial appearance which need correction to varying degrees by surgical intervention, speech therapy, dental treatment and psychosocial intervention(2). Thus in present case cleft lip and cleft palate were associated with bilateral paramedian lip pits, hypodontia, hypoplasia of teeth and shrunken uvula, which represents a syndromic form of OFC.

## Case Report

**Figure 1:**
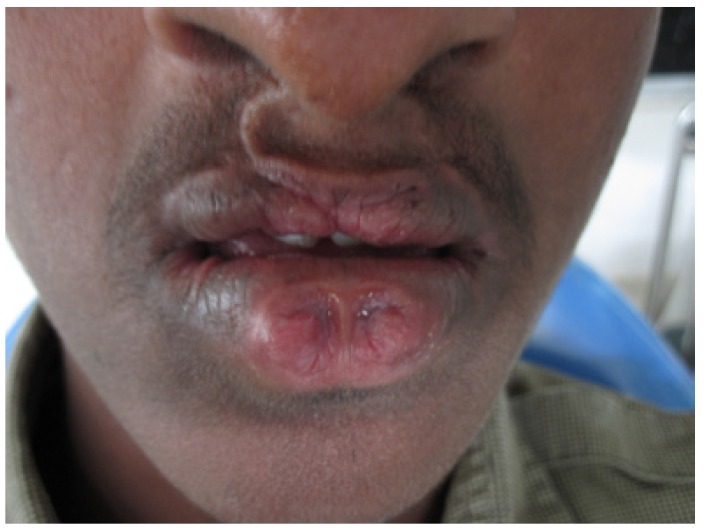
Extra oral features of patient showing the bilateral paramedian lower lip pits and the surgically repaired cleft of upper lip.

**Figure 2:**
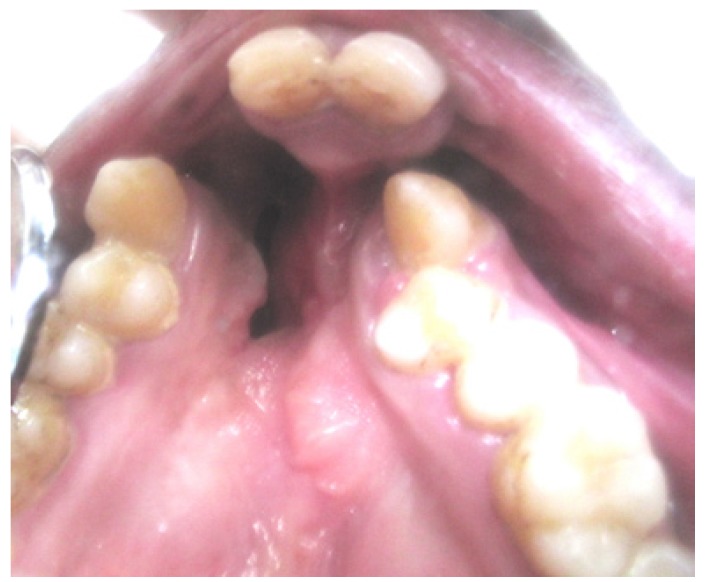
Intraoral features of patient showing the cleft palate with missing lateral incisors.

**Figure 3:**
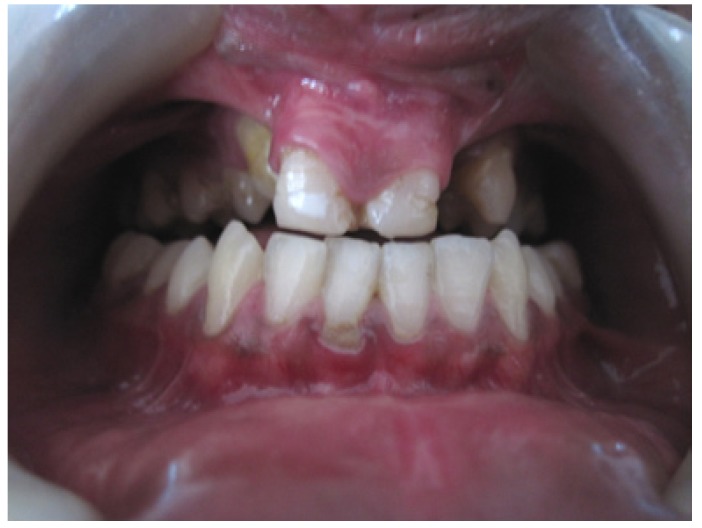
Intraoral features of patient showing the cross bite of arches and evident enamel hypoplasia of teeth.

A 19 year old male patient reported to the outpatient department of the department of Oral medicine and Radiology, Vishnu dental college, Bhimavaram, with the complaint of missing teeth in upper front tooth region. His past medical history was uneventful except for a surgical intervention at the age of 4 years for correction of lip abnormality. There was no consanguinity in the parents, and he has two unaffected siblings. The family history was negative for lip pits, clefts, and other congenital anomalies. Extra oral examination revealed mid face retrusion and a repaired bilateral cleft of the upper lip. His lower lip had bilateral lip pits (paramedian) of 3 mm diameter with continuous secretion of saliva (fig. 1). Intraoral examination revealed cleft palate with oronasal communication, with frequent complaint of regurgitation (fig. 2). Maxillary arch showed congenitally missing lateral incisors with a well aligned mandibular arch. Analysis of his occlusion revealed an anterior cross bite (fig. 3). Important variations were observed in oral cavity as hypoplasia of teeth and shrunken uvula in addition to the cardinal features of the syndrome (lip pits, cleft lip and cleft palate).

Whole body physical examination was then performed to rule out other condition having similar presentation (Popliteal pterigium syndrome, Oro-faciodigital syndrome). No other abnormality was detected thus a diagnosis of VWS was made. Radiographic investigation were performed (intraoral periapical radiograph, occlusal view of maxilla, postero-anterior and lateral skull views) for knowing any impacted teeth, jaw pathologies and also for treatment planning. Multidisciplinary approach for correction of clefts and complete rehabilitation was planned. Patient referred to department of oral maxillofacial surgery for excision of lip pits and tracts to avoid cosmetic problems and further complications from chronic inflammatory process of these lip pits. Followed by surgery, replacement for missing teeth and further management was advised. Importance of genetic counseling which is highly recommended as it provides information on likely hood of gene transmission and possible ways of expression was explained and advised to the patient.

## Discussion

When the labial pits occur in association with cleft lip and/or palate the condition is referred to as van der Woude Syndrome (VWS) (4). These cardinal clinical features of van der Woude syndrome are well documented in the literature (5). Hypodontia is considered as cardinal associated feature in 10-81% of all VWS patients with number of teeth missing in the upper jaw almost double that in corresponding control group(5). In the present case patient had a missing maxillary lateral incisor on either side and two important observations were made in addition to the clinical features as enamel hypoplasia of teeth and bulbous shrunken uvula. This kind of variable clinical presentation is the key feature of syndromic form of OFC.

Lip pits are inherited as an autosomal dominant trait, their pathogenesis is not well understood (5). They are thought to develop from notching of the lip at an early stage of the labial development with fixation of the tissue at the base of the notch or from failure of a complete union of the embryonic lateral sulci of the lip, which persist and ultimately develop into the typical pits(5). A single median or paramedian lip pit is considered as incomplete expression of trait whereas bilateral lip pits are features of complete expression (6). Thus in present case bilateral paramedian lip pits are present denoting the complete expression of this condition.

van der Woude syndrome has an autosomal dominant hereditary pattern with a variable expressivity, and its penetrance has been estimated at 80%, however later reports have shown that the penetrance is close to 100% (7). Eighty percent of gene carriers are not diagnosed because they are nonpenetrant, among the penetrant gene carriers, however, as many as 80% may not have been recognized in the past (7). The VWS loci were initially mapped to human chromosome 1q32–q41 and phenotypes were subsequently demonstrated to result from mutations in the gene encoding interferon regulatory factor 6 (IRF6) (2). IRF6 belongs to a family of transcription factors that share a highly conserved N-terminal, penta-tryptophan, helix-turn-helix DNA-binding domain and a less well-conserved protein-binding domain(2). The risks of inheriting a cleft from an affected parent is 22.43%, whereas the risk of inheriting lip pits only or being nonpenetrant is 27.57% (8). Affected males and females are equally likely to transmit VWS (7). The differential diagnosis should include Popliteal pterigium syndrome (popleteal webs, cleft lip/palate, lower lip pits, anomalies of genitor-uterine tract as cryptorchidism and bifid scrotum in males, hypoplastic labia majora and uterus in females) and Oro-facio-digital syndrome (orofacial features of cleft palate, bifid tongue, hypodontia, lip pits etc. with digital, renal and central nervous system abnormality (5). Which are considered to be much complicated than the van der Woudes syndrome.

Management of VWS is majorly focused on surgical and cosmetic correction of clefts and lip pits, on account of secondary infection due to inflammation of pits may necessitate usage of antibiotic, analgesics and meticulous oral hygiene practices (5). More extreme phenotypes in parents tend to produce more extreme expression in their children. However, the lesser expressions of VWS are common and should be actively looked for when counseling families about cleft lip or cleft lip and palate (7). As observed in this case a variable presentation with complete expression, patients was advised genetic counseling and benefits from it. With these considerations in mind all patients should be informed of the 50% inheritance of this disorder and genetic counseling encouraged (7). The potential of embryoscopy to detect minor malformations such as cleft lip allows for early termination of pregnancy in patients with VWS (9). Furthermore a case of squamous cell carcinoma was reported as developing from chronically inflamed lip pit (5) which cautions us for thorough evaluation, early intervention and treatment of this condition.

## Conclusion

van der Woude syndrome is not frequently reported, the phenomenon of cleft lip and cleft palate combined in same pedigree makes it unique. Making a distinction between similar disease presenting cases requires thorough knowledge of various clinical features, variable manifestations and investigatory procedures. A multidisciplinary approach including genetic counseling which would give information on likelihood of gene transmission, possible ways of expression and the potential of embryoscopy to detect minor malformations such as cleft should be encouraged in early diagnosis and management.
